# Thermodynamic Properties of 3- and 4-Ethoxyacetanilides between 80 and 480 K

**DOI:** 10.3390/molecules28207027

**Published:** 2023-10-11

**Authors:** Andrey A. Sokolov, Mikhail I. Yagofarov, Ilya S. Balakhontsev, Ilyas I. Nizamov, Timur A. Mukhametzyanov, Boris N. Solomonov, Yana N. Yurkshtovich, Elena N. Stepurko

**Affiliations:** 1 Department of Physical Chemistry, Kazan Federal University, Kremlevskaya Str. 18, 420008 Kazan, Russia; miiyagofarov@kpfu.ru (M.I.Y.); jsyoutub@gmail.com (I.S.B.); ilyinizamov@stud.kpfu.ru (I.I.N.); timur.mukhametzyanov@kpfu.ru (T.A.M.); 2 Chemistry Department, Belarusian State University, 220030 Minsk, Belarus; yanayurksht@gmail.com (Y.N.Y.); stepurko@bsu.by (E.N.S.)

**Keywords:** adiabatic calorimetry, differential scanning calorimetry, fast scanning calorimetry, solution calorimetry, heat capacity, fusion enthalpy, amides, phenacetin, 3-ethoxyacetanilide

## Abstract

In this work, we present a comprehensive study of the thermodynamic properties of 3-and 4-ethoxyacetanilides. The heat capacities in crystalline, liquid, and supercooled liquid states from 80 to 475 K were obtained using adiabatic, differential scanning (DSC), and fast scanning (FSC) calorimetries. The fusion enthalpies at *T*_m_ were combined from DSC measurement results and the literature data. The fusion enthalpies at 298.15 K were evaluated in two independent ways: adjusted according to Kirchhoff’s law of thermochemistry, and using Hess’ law. For the latter approach, the enthalpies of the solution in DMF in crystalline and supercooled liquid states were derived. The values obtained by the two methods are consistent with each other. The standard thermodynamic functions (entropy, enthalpy, and Gibbs energy) between 80 and 470 K were calculated.

## 1. Introduction

The investigation of compounds containing an amide bond is valuable since it makes up peptide and protein structures [1]. It is of great interest to study the thermodynamic properties of unsubstituted amides [2,3] and their more complex derivatives [4,5,6,7] for both fundamental and applied science purposes.

**Figure 1:**
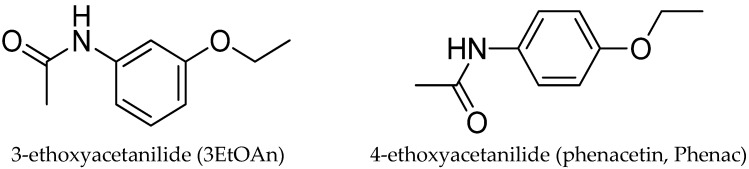
The structural formulas of 3-ethoxyacetanilide and phenacetin.

In this work, we focused on 3- and 4-ethoxyacetanilides (Figure 1). 4-ethoxyacetanilide (Phenac), also known as phenacetin, has been long used in medicine as an analgesic and antipyretic drug [8,9], and despite not being prescribed now due to side effects [10,11,12,13], it is still utilized widely as a pharmaceutical standard [14,15,16]. 3-ethoxyacetanilide (3EtOAn) is isomeric to phenacetin and has twice the antipyretic effect [17].

The thermodynamic functions of acetanilide derivatives, including ethoxyacetanilides, have been studied in a number of recent papers, particularly devoted to the thermochemistry of fusion [3,7,18,19,20,21,22], vaporization [3,7], sublimation [3,7,23], dissolution in water [24], as well as solubility in neat and binary solvents [4,25,26,27]. Despite a large number of studies, some thermodynamic quantities, such as condensed state heat capacities and enthalpies of fusion at 298.15 K, remain unknown, which complicates the determination and verification of other thermodynamic parameters. For example, the difference between the heat capacities of a crystal and a liquid is necessary for the correct calculation of the ideal solubility and the elimination of the systematic errors from the approaches for activity coefficients’ estimation [28]. The independent determination of the fusion enthalpy at 298.15 K would be advantageous for establishing the consistency between the thermochemical parameters of vaporization and sublimation at 298.15 K.

In this work, the heat capacities of these compounds in a wide temperature range in crystal, liquid, and supercooled liquid states were studied using a combination of methods, including adiabatic, differential scanning (DSC), and fast scanning (FSC) calorimetry. The fusion enthalpies at their melting point were measured by DSC and adjusted to 298.15 K with the obtained heat capacities.

The validity of the fusion enthalpy values at 298.15 K was additionally verified using Hess’ law and data on the solution enthalpies of the studied compounds in DMF. Finally, the standard thermodynamic functions (entropy, enthalpy, and Gibbs energy) were derived in the temperature range from 80 to 470 K.

## 2. Results

### 2.1. Fusion Enthalpies of 3-Ethoxyacetanilide and Phenacetin at Melting Temperature

The fusion enthalpies of 3EtOAn and Phenac, available from the literature, are presented in Table 1. Twelve fusion enthalpy values are available for Phenac, and only a single value is reported for 3EtOAn.

The fusion enthalpy of 3EtOAn reported by Umnahanant et al. [3] (28.9 ± 0.4 kJ mol^−1^) is in good agreement with the one obtained in this work (28.1 ± 0.5 kJ mol^−1^, see Table S2), so the arithmetic mean of these values (28.5 ± 0.6 kJ mol^−1^) was used in further calculations.

As for Phenac, the literature data vary within the range of 15 kJ mol^−1^. After excluding the outliers (21.4 and 36.9 kJ mol^−1^), the range reduces to 5 kJ mol^−1^, remaining significant. The mean fusion enthalpy is 31.2 ± 1.7 kJ mol^−1^, agreeing with the value obtained in this work (31.2 ± 0.5 kJ mol^−1^).

### 2.2. Heat Capacity and Thermodynamic Properties of 3-Ethoxyacetanilide between 80 and 430 K and Phenacetin between 80 and 475 K

The experimental heat capacities of 3EtOAn and Phenac, determined by adiabatic calorimetry in the temperature ranges of (80 to 369) K and (80 to 367) K, respectively, are shown in Tables S3 and S4 of the Supplementary Materials. For 3EtOAn, in the region above 352 K, an anomalously fast increase in heat capacity with an increasing temperature is observed, which corresponds to the onset of the melting of the substance. The upper temperature limit of measurements of the adiabatic calorimeter (370 K) makes it possible to fix only part of the ascending branch of the melting curve and does not allow the determination of the fusion enthalpy of the substance; therefore, these values were not used in the further calculations.

**Figure 2:**
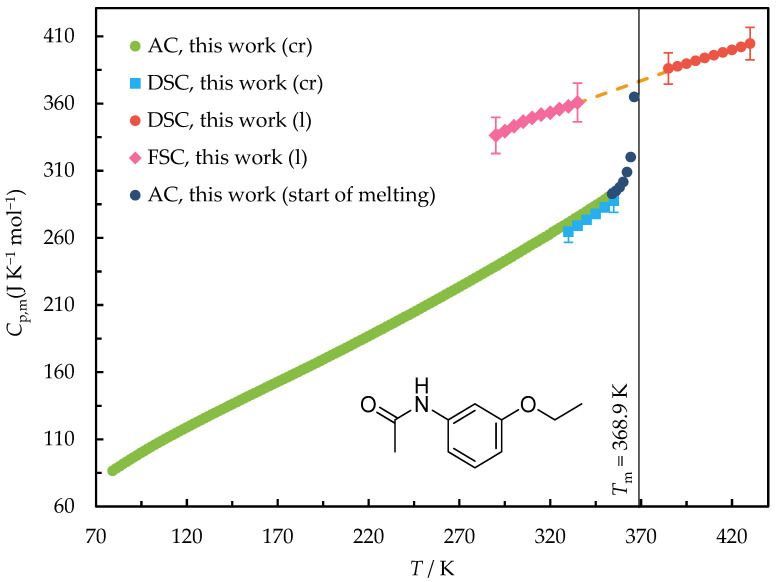
The heat capacities of 3EtOAn measured by adiabatic calorimetry (AC), DSC, and FSC in the temperature range of (80 to 430) K. The orange dashed line is the polynomial approximation of the heat capacity of liquid.

**Figure 3:**
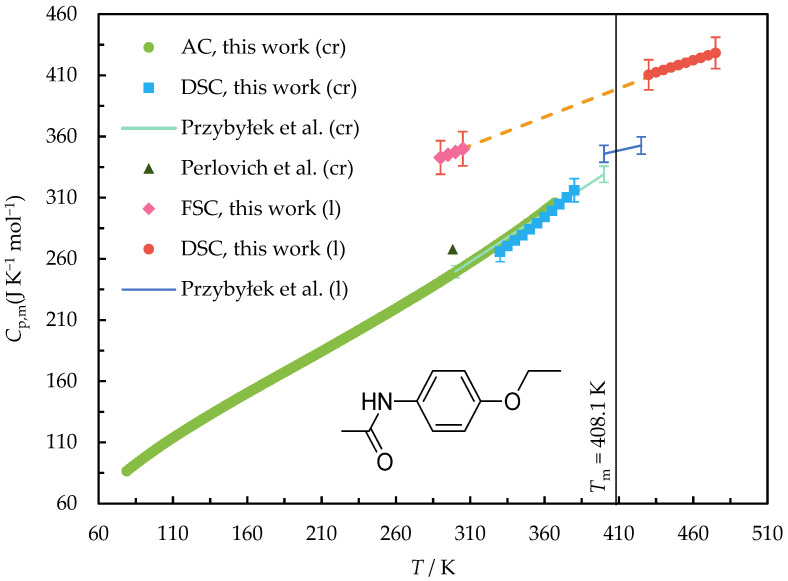
The heat capacities of Phenac measured by adiabatic calorimetry (AC), DSC, and FSC in the temperature range of (80 to 475) K and available from Refs. [22,34]. The orange dashed line is the polynomial approximation of the heat capacity of liquid.

The heat capacities measured by DSC and FSC in the temperature ranges of (290 to 430) K for 3EtOAn and (290 to 475) K for Phenac are given in Table S5. A graphic representation of the obtained data is shown in Figure 2 and Figure 3.

The experimental curves of the DSC and FSC measurements of the fusion enthalpies and heat capacities are shown in Figures S1–S3 of the Supporting Information.

A very good agreement was observed between *C*_p,m_(cr, *T*) determined by DSC and adiabatic calorimetry in the overlapping temperature ranges of (330–350) K and (330–365) K for 3EtOAn and Phenac, respectively.

The extrapolation of the heat capacity values measured by DSC to the temperature range below the melting point corresponds to the heat capacities determined by FSC within 3%. Thus, the values produced by both DSC and FSC were used for deriving the liquid state heat capacity.

The heat capacities of Phenac in the crystal and supercooled liquid states were previously determined using DSC by Przybyłek et al. [22]. The values of the heat capacity of the crystal agree with the data measured in this work by adiabatic calorimetry within 1% in the overlapping temperature range. On the other hand, the heat capacity of the supercooled liquid Phenac extrapolated from Ref. [22] is 14–15% lower than that determined in this work. Since the temperature range of the liquid state heat capacity measurements in Ref. [22] is less than 20 K, only the data obtained in our work were used in the further calculations.

The heat capacity of the solid Phenac at a single temperature of 298.15 K was measured by Perlovich et al. [34]. Its value is 7.5% higher than both the value from Ref. [22] and that determined in the present work.

The thermodynamic properties (Table 2 and Table 3) were derived using the polynomial approximations of the experimental heat capacities. The coefficients and temperature ranges are reported in Tables S7 and S8 of the Supplementary Materials. The relative deviations between the experimental and approximated values (Δ = *C*_p,m_(exp)/*C*_p,m_ (app) − 1) are shown in Figures S4–S7.

In the temperature range near the melting temperature, the heat capacities were described with linear functions:

For 3EtOAn:

*C*_p,m_(cr, *T*)/(J K^−1^ mol^−1^) = −12.12 + 0.8587 (*T*/K); 325 < T/K < 352 (1)

*C*_p,m_(l, *T*)/(J K^−1^ mol^−1^) = 200.1 + 0.4780 (*T*/K); 289 < T/K < 430 (2)

For Phenac:

*C*_p,m_(cr, *T*)/(J K^−1^ mol^−1^) = −30.88 + 0.9176 (*T*/K); 346 < T/K < 367 (3)

*C*_p,m_(l, *T*)/(J K^−1^ mol^−1^) = 207.3 + 0.4685 (*T*/K); 290 < T/K < 477 (4)

## 3. Discussion

### 3.1. Heat Capacities of the Solid and Liquid 3-Ethoxyacetanilide and Phenacetin at 298.15 K

The determination of the precise values of the condensed-phase heat capacities in a wide temperature range is a non-trivial task and requires combining several experimental techniques. In this regard, numerous predictive schemes have been developed. A comparison of the estimated values with the results of this work will help to understand the possibility of their application for obtaining the heat capacities of acetanilide derivatives.

Chickos et al. developed a first-order group contribution approach that enables the calculation of the liquid and solid heat capacities at 298.15 K for a wide range of organic compounds [35].

The estimation of the solid heat capacity at 298.15 K was performed with a simple element-based additivity scheme by Hurst et al. [36]. The heat capacity of crystal as a function of temperature can be estimated using two methods proposed by Goodman et al. [37]. The first one (PL) is based on an empirical temperature dependence of the heat capacity in a power-law form, while the second one (PF) utilizes the Einstein–Debye partition function and requires knowledge or the estimation [38,39] of the radius of gyration.

Several second- and third-order group contribution schemes have been proposed for the prediction of the liquid heat capacity as a function of temperature. The most popular and precise is the one developed by Ruzicka, Domalski [40,41], and Zabransky [42], and further improved by Kolska [43].

In Table 4, the experimental heat capacities of liquid and solid 3EtOAn and Phenac at 298.15 K, as well as the values estimated using the above-mentioned schemes, are summarized.

Surprisingly, the simplest element-based method of Hurst et al. gave the most accurate results in predicting the heat capacity of the solid. The application of Goodwin et al.’s schemes leads to underestimated values, especially with the more advanced PF method. In contrast, the additive scheme of Chickos et al. overestimated the heat capacity of the crystal by 13–15%.

The opposite situation is observed in the case of the heat capacity of the liquid, where Chickos’ method gives values slightly higher than the experimental results. The values closest to the experimental heat capacities are those obtained using the scheme by Kolska et al.

It may be concluded that the prediction of the heat capacities of ethoxyacetanilides in the condensed phase using additive methods can lead to significant errors. This could be associated with the presence of hydrogen bonding or the lack of such compounds in the databases on which the schemes are built.

### 3.2. The Difference between the Heat Capacities of the Solid and Liquid 3-Ethoxyacetanilide and Phenacetin at the Melting Point

Another important thermodynamic property that can be found from the data obtained in this work is the difference between the liquid and solid heat capacities, $\Delta_{\mathrm{cr}}^{l}C_{p,m}^{A}$. In the case of pharmaceuticals, it is particularly often used when calculating ideal solubility, according to the Schroeder–Le Chatelier equation [44]:

(5) $$\begin{array}{c} \ln x_{\mathrm{id}}^{A}(T)=\frac{\Delta_{\mathrm{cr}}^{l}H^{A}(T_{m})}{R}(\frac{1}{T_{m}}-\frac{1}{T})+\\ +\frac{\int_{T_{m}}^{T}\Delta_{\mathrm{cr}}^{l}C_{p,m}^{A}(T^{\prime })\;dT^{\prime }}{RT}-\frac{\int_{T_{m}}^{T}\frac{\Delta_{\mathrm{cr}}^{l}C_{p,m}^{A}(T^{\prime })}{T^{\prime }}\;dT^{\prime }}{R} \end{array}$$

Since the determination of the $\Delta_{\mathrm{cr}}^{l}C_{p,m}^{A}$ value in the entire temperature range of application is obstructed by the crystallization of the supercooled liquid, various approaches are used to estimate $\Delta_{\mathrm{cr}}^{l}C_{p,m}^{A}$. It is often neglected [45,46,47], but this can lead to substantial errors [28]. When experimental $\Delta_{\mathrm{cr}}^{l}C_{p,m}^{A}$ at *T*_m_ is available, it is frequently presumed to be temperature-independent [48,49]. There are many compounds for which such an assumption is incorrect [50]. Otherwise, several approaches have been developed for the estimation of this value. Their performance was tested in relation to the prediction of the $\Delta_{\mathrm{cr}}^{l}C_{p,m}^{A}(T_{m})$ value for 3EtOAn and Phenac.

The earlier ones took $\Delta_{\mathrm{cr}}^{l}C_{p,m}^{A}$ to be equal to the fusion entropy [51,52,53,54]. In Ref. [44], Pappa et al. considered three schemes for the calculation of the difference between the liquid and solid heat capacities of the organic compounds at the melting temperature.

Scheme I is based on the group additivity approaches for the calculation of the solid and liquid heat capacities, as discussed earlier. Its variations, Ia and Ib, correspond to the PL and PF methods of Goodman et al.’s [37] scheme for a solid heat capacity prediction. The heat capacity of the liquid was originally calculated using the Ruzicka and Domalski group contribution approach [40,41], but we implemented the method by Kolska et al. [43] since it has a lower relative error.

Scheme II uses the assumption that the difference between the solid and liquid heat capacities at the melting point is equal to the fusion entropy: $\Delta_{\mathrm{cr}}^{l}C_{p,m}^{A}(T_{m})\;=\;\Delta S_{f}^{A}(T_{m})$.

Scheme III implies a correlation between $\Delta_{\mathrm{cr}}^{l}C_{p,m}^{A}(T_{m})$ and $\Delta S_{f}^{A}(T_{m})$, which varies for different classes of organic substances. The parameters of the correlation for the amides are not presented in Ref. [44], therefore, it is impossible to utilize this scheme for the studied compounds.

Wu and Yalkowsky developed an equation that allows for the calculation of $\Delta_{\mathrm{cr}}^{l}C_{p,m}^{A}(T_{m})$ using such structural parameters as the molecular flexibility number, the molecular symmetry number, and the hydrogen bond number [55].

The experimental and estimated values of the difference between the solid and liquid heat capacities and the resulting ideal solubilities are summarized in Table 5.

One can see that the $\Delta_{\mathrm{cr}}^{l}C_{p,m}^{A}$ values predicted by different approaches vary by more than two times. In turn, there is a more than two-fold variation in the *x*_id_ predicted using $\Delta_{\mathrm{cr}}^{l}C_{p,m}^{A}=0$ and Pappa, 1b approximations in the case of Phenac and 40% variation for 3EtOAn. The greater variation in the case of Phenac is due to its 39 K-greater *T*_m_. In the case of the ethoxyacetanilides considered in this work, the neglect of the temperature dependence of $\Delta_{\mathrm{cr}}^{l}c_{p,m}^{A}$ does not contribute significantly to the calculations according to Equation (5).

### 3.3. Fusion Enthalpies of 3-Ethoxyacetanilide and Phenacetin at 298.15 K

Considering the substantial inconsistency of the available data on the fusion enthalpy of Phenac and the insufficient amount of data on the thermochemical properties of 3EtOAn, it is necessary to validate the values obtained in this work.

One can do this by comparing the enthalpies of fusion at 298.15 K obtained in two independent ways.

On the one hand, the enthalpy of fusion at the melting temperature can be adjusted to 298.15 K using Kirchhoff’s law of thermochemistry and the heat capacities of the crystal and liquid phases measured in this work:

(6) $$\Delta_{\mathrm{cr}}^{l}H_{}^{A}(298.15\;K)=\Delta_{\mathrm{cr}}^{l}H_{}^{A}(T_{m})+\int_{T_{m}}^{298.15}\Delta_{\mathrm{cr}}^{l}C_{p,m}^{A}(T)\;dT$$

where $\Delta_{\mathrm{cr}}^{l}H_{}^{A}(298.15\;K)$ and $\Delta_{\mathrm{cr}}^{l}H_{}^{A}(T_{m})$ are the enthalpies of the fusion of compound A at 298.15 K and *T*_m_, respectively, and $\Delta_{\mathrm{cr}}^{l}C_{p,m}^{A}(T)$ is the difference between the liquid and crystal molar heat capacities of compound A at temperature *T*.

For the calculation of Kirchhoff’s integrals, the values of the crystal heat capacities determined by adiabatic calorimetry and the values of the liquid heat capacities obtained by a combination of DSC and FSC were used. By combining the integrals and enthalpies of fusion at the melting temperature presented in Table 1, the following fusion enthalpies at 298.15 K can be derived: 22.4 ± 1.2 kJ mol^−1^ for 3EtOAn and 22.5 ± 2.2 kJ mol^−1^ for Phenac.

On the other hand, it is also possible to apply Hess’ law to the processes of fusion and the solution of the compound, i.e.,

(7) $$\Delta_{\mathrm{cr}}^{l}H_{}^{A}(298.15\;K)=\Delta_{\mathrm{soln}}^{}H_{}^{A/S}(\mathrm{cr},\;298.15\;K)-\Delta_{\mathrm{soln}}^{}H_{}^{A/S}(l,\;298.15\;K)\;$$

where the solution enthalpy of crystal A in solvent S at 298.15 K $\Delta_{\mathrm{soln}}^{}H_{}^{A/S}(\mathrm{cr},\;298.15\;K)$ is measured experimentally, while the solution enthalpy of the supercooled liquid in the same solvent S at 298.15 K $\Delta_{\mathrm{soln}}^{}H_{}^{A/S}(l,\;298.15\;K)\;$ is not directly available, but can be often estimated. It is convenient to use a solvent that is structurally similar to the compound of interest, i.e., the one in which the solution enthalpy of the liquid is close to zero or can be found using solute-like compounds. For example, we previously showed that the enthalpy of the solution of non-hydrogen-bonded aromatic liquids in benzene ranges from 0 to 2 kJ mol^−1^ and is 1 ± 1 kJ mol^−1^ on average [56]. For liquid hydrogen-bonded aromatic compounds, the solvents with a similar basicity can be used (anisole for phenol derivatives, aniline for aromatic amines, methyl benzoate for benzoic acid derivatives [57]). For polyols such as D-sorbitol, D-mannitol, and myo-inositol, a suitable solvent is water, in which the amorphous phase of these compounds dissolves with a near-zero thermal effect, as demonstrated in Ref. [58].

Applying a similar approach, in Ref. [59], the solution enthalpies of liquid benzamide, *N*-methylbenzamide, and acetanilide in dimethylformamide (DMF) were evaluated using the solution of liquid aliphatic amides, *N*-methylformamide, and formamide. The main idea of this concept was that the solution enthalpy of amides in DMF, $\Delta_{\mathrm{soln}}^{}H_{}^{A/\mathrm{DMF}}(l)$, mainly depends on differences in the hydrogen bonding between the solute and DMF, $\Delta_{\mathrm{HB}}^{}H_{}^{A/\mathrm{DMF}}$, and the solute itself, $\Delta_{\mathrm{HB}}^{}H_{}^{A/A}$:

(8) $$\Delta_{\mathrm{soln}}^{}H_{}^{A/\mathrm{DMF}}(l)\approx \Delta_{\mathrm{HB}}^{}H_{}^{A/\mathrm{DMF}}-\Delta_{\mathrm{HB}}^{}H_{}^{A/A}$$

It was found that for acetanilide, the solution enthalpy of the liquid in DMF was equal to −4.3 kJ mol^−1^. The introduction of the ethoxy group should not significantly affect the acidity and basicity of the NHCO group. The slight changes present are compensated according to Equation (7). Therefore, we assumed that $\Delta_{\mathrm{soln}}^{}H_{}^{A/\mathrm{DMF}}(l)$ = −4.3 ± 1.0 kJ mol^−1^ for both investigated substances.

For the evaluation of the fusion enthalpy of 3EtOAn and Phenac at 298 K using Hess’ law, the solution enthalpy of the solid is also required. The average values of $\Delta_{\mathrm{soln}}^{}H_{}^{A/\mathrm{DMF}}(\mathrm{cr})$ found in this work were 19.02 ± 0.23 kJ mol^−1^ for 3EtOAn and 20.60 ± 0.06 kJ mol^−1^ for Phenac; the experimental values are provided in Table S6.

Thus, the fusion enthalpies of 3EtOAn and Phenac at 298.15 K obtained using solution calorimetry are 23.3 ± 1.0 kJ mol^−1^ and 24.9 ± 1.0 kJ mol^−1^, respectively. In both cases, the agreement with Kirchhoff’s law of thermochemistry is within the combined uncertainty. The greater deviation in the case of 4-ethoxyacetanilide, whose *T*_m_ is notably higher, can be due to the greater contribution of the error of the liquid state heat capacity extrapolation.

The consistency between the fusion enthalpies obtained by Kirchhoff’s and Hess’ laws validates the previous estimates of the solution enthalpies of supercooled liquid aromatic amides in DMF, i.e., the ethoxy group in the benzene ring is likely to have little effect on the hydrogen bonding strength of acetanilides with DMF.

The fusion enthalpies of the studied compounds at *T*_m_ and 298.15 K are presented in Table 6.

## 4. Materials and Methods

### 4.1. Materials

3-ethoxyacetanilide (3EtOAn, Alfa, CAS No. 591-33-3), 4-ethoxyacetanilide (phenacetin, Phenac, Alfa, CAS No. 62-44-2) (Figure 1), and *N,N*-dimethylformamide (DMF, Aldrich, CAS No. 68-12-2) were of commercial origin with initial mass fraction purities greater than 0.999. *N*,*N*-dimethylformamide was stored over molecular sieves (4 Å). The water content in *N,N*-dimethylformamide was checked by Karl Fischer titration using an automatic titrator C20 (Mettler Toledo) and HYDRANAL™ as a reagent. Solid samples were dried in vacuo before the measurement. The information is summarized in Table S1 of the Supplementary Materials.

### 4.2. Adiabatic Calorimetry

The heat capacities of 3EtOAn and Phenac under vapor saturation pressure ($C_{s,m}$) in the temperature range of (79–369) K were determined using a Termis TAU-10 automatic vacuum adiabatic calorimeter, according to previously reported experimental procedure [60]. The temperature was measured with an Fe–Rh resistance thermometer (R = 50 Ω, calibrates on ITS-90 by VNIIFTRI, Moscow, Russia) with the standard uncertainty of 0.01 K. The adiabatic conditions in the calorimeter were maintained using a differential thermocouple (Cu + 0.1% Fe)/chromel and two heaters, i.e., the main one and the additional one, to eliminate temperature gradients over the length of the adiabatic shell. Heater control and visible energy detection, temperature measurements, and calculations of heat capacities in the calorimetric experiment were performed using the AK-6.25 automatic control unit.

Titanium calorimetric cell (V = 1.13 cm^3^) was loaded in the air with solid substances of 0.6395 g (3EtOAn) and 0.6872 g (Phenac) up to 4/5 of its volume and was degassed in the vacuum for 30 min. After the degassing stage, container was filled with helium at 10 kPa, and then it was hermetically sealed with a bronze lid to provide rapid establishment of thermal equilibrium during calorimetric measurements. Indium ring was used as a sealant. Container with a sample was weighed on a Mettler-Toledo AG245 balance (with a maximum error of ±5 × 10^−5^ g), and the hermiticity of the container was controlled by several cycles of exposure in the air and in vacuum to achieve fixed mass. Liquid nitrogen was used as a cooling agent in the temperature range of (77 to 370) K. The relative expanded uncertainty (0.95 level of confidence for normal distribution, k ≈ 2) of the heat capacity measurements in the TAU-10 was 0.4% in the mentioned temperature range. The share of the sample’s heat capacity in the total heat capacity of the filled calorimetric ampoule was (0.33 to 0.42) in the temperature range of (79 to 352) K for 3EtOAn and (0.35 to 0.45) in the temperature range of (79 to 369) for Phenac. The difference between heat capacities at the saturation pressure $C_{s,m}$ and molar isobaric heat capacities $C_{p,m}^{o}$ was found to be negligible in comparison with experimental uncertainty in the studied temperature range (i.e., $C_{s,m}$ ≈ $C_{p,m}^{o}$). The heating period was 400 s. The thermal relaxation was observed for (280 to 300) s. The temperature drift was measured for 400 s. The temperature step was approximately 2 K. Experimental results from adiabatic calorimetry measurements are presented in the Supplementary Materials (see Tables S3 and S4).

### 4.3. Differential Scanning Calorimetry

The molar heat capacities (*C*_p,m_), enthalpies, and temperatures of fusion ($\Delta_{\mathrm{cr}}^{l}H(T_{m})$) of Phenac and 3EtOAn were measured using DSC 8500 (Perkin Elmer, Waltham, MA, USA). Reference Indium and Zinc samples were used for DSC calibration, as recommended by the manufacturer. After the calibration, $\Delta_{\mathrm{cr}}^{l}H(T_{m})$ and *C*_p,m_ of bismuth, benzoic acid, and anthracene were measured to check DSC performance, as previously [61]. The information on the samples’ origin and purity is provided in Table S1. The reference thermochemical data on these compounds were available from Refs. [62,63]; the obtained quantities agreed with the literature data within 2%.

Before the measurement, aluminum crucibles (50 μL) were heated to 473 K to remove the traces of moisture. Glovebox filled with argon was used to place the samples in the crucibles. Two samples of each compound were studied. Both heat capacity and fusion enthalpy measurements were repeated twice on each sample. Heating and cooling rates were ±10 K min^−1^. Experiments were performed in a dynamic nitrogen (Nurgas, volume fraction 0.99999, dew point < 173 K) atmosphere (30 mL min^−1^).

For $\Delta_{\mathrm{cr}}^{l}H(T_{m})$ determination, the samples were cycled between the room temperature and (*T*_m_ + 40 K). Both compounds crystallized completely on cooling at 10 K min^−1^. Experimental results from DSC measurements are presented in the Supplementary Material (see Table S5).

The *C*_p,m_ measurements were performed in the temperature ranges (*T*_min_–*T*_max_) of 330–355 K and 385–430 K for crystal and liquid 3EtOAn, respectively, and 330–380 K and 430–475 K for crystal and liquid Phenac.

The three-step procedure for the heat capacity measurement was applied. The first step was the baseline determination for the empty crucibles. The temperature program included the dynamic segments and two isothermal segments at the lowest and highest temperatures of the measurements. Applying the same program, a standard sample (sapphire) (*m* = 33.79 mg) and a sample of the studied compound (10 < *m*/mg < 20) were sequentially measured in the same crucible.

The reproducibility of heat flow and temperature calibration (0.95 level of confidence, *k* ≈ 2) was equal to 1% and 0.1 K, respectively. The reproducibility (0.95 level of confidence, *k* ≈ 2) of the heat capacities was within %. These values were included in the propagated error of the heat capacity measurement, which equaled 3% in total. Pyris 13 Software was used to calculate *C*_p,m_ from the calorimetric curves.

### 4.4. Fast Scanning Calorimetry

The heat capacities of supercooled liquid 3EtOAn between 290 and 335 K and of Phenac between 290 and 305 K were determined using Mettler Toledo Flash DSC2+ (Mettler Toledo, Greifensee, Switzerland). Preliminarily temperature calibration of the UFS1 chip sensors was performed using biphenyl, benzoic acid, and anthracene samples. The temperature calibration was reproducible within ±2 K (expanded uncertainty at 0.95 level of confidence, *k* ≈ 2). The experiments were performed in a dynamic nitrogen (Nurgas, volume fraction 0.99999, dew point < 173 K) atmosphere (40 mL min^−1^). The sensor support temperature and the minimal measurement temperature were 183 K. The diameter of the measuring area of the UFS1 chip sensors was 0.5 mm, so small samples of 40–300 ng were studied. The scanning rates *β* = ±1000 K s^−1^ were used.

*C*_p,m_ of the supercooled liquid was determined as follows. In the absence of phase transitions and chemical processes, the heat flow rate on cooling and heating, *Φ*(W), (corrected for the heat flow to the empty sensor) is a sum of the sample heat capacity contribution (*C*_p_(*T*)*β*, with *C*_p_(*T*) being an absolute heat capacity of the sample) and the heat losses (*Φ*_HL_) [64]:

Heating:

(9) $$\Phi_{h}(T)=nC_{p,m}(T)\beta_{h}+\Phi_{\mathrm{HL},h}(T)$$

Cooling:

(10) $$\Phi_{c}(T)=nC_{p,m}(T)\beta_{c}+\Phi_{\mathrm{HL},c}(T)$$

For equal heating and cooling rates *β*_h_ = *β*_c_ = *β*, the *Φ*_HL,h_ and *Φ*_HL,c_ can be assumed to be equal as well. Then the absolute heat capacity of the sample can be found according to Equation (11):

(11) $$C_{p}(T)=\frac{\Phi_{c}(T)-\Phi_{h}(T)}{2\beta }$$

From the absolute heat capacity of the crystal and its molar heat capacity, determined by adiabatic calorimetry, the sample amount can be found: *n* = *C*_p_(cr, *T*)/*C*_p,m_(cr, *T*). Then, vice versa, *C*_p,m_(l, *T*) is found as *C*_p_(l, *T*)/*n.* The combined uncertainty of *C*_p,m_(l, *T*) determined in this way is below 4% (0.95 level of confidence, *k* ≈ 2).

Five samples of 3EtOAn and Phenac were measured. After the placement on the sensor, the samples were heated to *T*_m_ + 5 K and rapidly cooled at 5000 K s^−1^. Such pre-melting procedure guarantees the reproducible thermal contact between the sensor and the recrystallized sample. *T*_m_ of 3EtOAn and Phenac after pre-melting were 368 ± 2 K and 408 ± 2 K, agreeing with those obtained using DSC.

To determine *C*_p_(cr, *T*) and *n* = *C*_p_(cr, *T*)/*C*_p,m_(cr, *T*), the samples were cycled three times between 183 K and *T*_max_ at *β* = ±1000 K s^−1^ with an isotherm (0.1 s) at 183 K. Then, the samples were heated to *T*_m_ + 20 K at *β* = 1000 K s^−1^, forming the liquid, and instantly cooled to 183 K. Then the samples were cycled between 183 K and *T*_max_ at *β* = ±1000 K s^−1^ again to determine *C*_p_(l, *T*) and *C*_p,m_(l, *T*) = *C*_p_(l, *T*)/*n*. *T*_max_ was 343 K for 3EtOAn and 348 K for Phenac. At the chosen *T*_max_, no sample mass losses were observed when comparing *Φ*_c_ and *Φ*_h_ on the consequential scans, as described in Ref. [64].

The scanning rates of ±1000 K s^−1^ were enough to prevent crystallization of 3EtOAn on cooling and heating. The half-step glass transition temperature (*T*_g_) at the heating rate *β* = +1000 K s^−1^ was 271 ± 2 K. Phenac did not crystallize on cooling, but exhibited a cold crystallization peak starting at *T* = 324 K at *β* = +1000 K s^−1^. Its *T*_g_ under analogous conditions was 278 ± 2 K. This prevented the determination of the heat capacity of supercooled liquid Phenac in a wider range.

### 4.5. Solution Calorimetry

The solution enthalpies of 3EtOAn and Phenac in *N,N*-dimethylformamide (DMF) were measured at 298.15 K using TAM III precision solution calorimeter (TA Instruments, New Castle, DE, USA). The compounds were dissolved by breaking a glass ampule filled with 30–40 mg of the studied sample into a glass cell containing 90 mL of pure solvent. Five samples of each compound were studied. The infinite dilution conditions were achieved, which was confirmed by the absence of the systematic variation of the solution enthalpy in the concentration range of 2.25 to 12.66 mmol kg^−1^. The correct operation of the calorimeter was previously validated by determining the solution enthalpies of propanol-1 in bidistilled water [59].

## 5. Conclusions

In this work, the fusion enthalpies and heat capacities of 3-ethoxyacetanilide and phenacetin in the crystalline, liquid, and supercooled liquid states were measured in a wide temperature range using a combination of adiabatic calorimetry, DSC, and FSC. The heat capacities obtained by the different methods agreed within 3%. These values, together with the literature data, were used to adjust the enthalpies of fusion from *T*_m_ to 298.15 K, according to Kirchhoff’s law of thermochemistry. The accuracy of these evaluations was additionally checked with solution calorimetry by determining the difference between the solution enthalpies of the solid and supercooled liquid in DMF. The difference in the enthalpies of fusion at 298.15 K, obtained by the two independent methods, did not exceed 3 kJ mol^−1^. The standard thermodynamic properties (entropy, enthalpy, and Gibbs energy) of 3-ethoxyacetanilide and phenacetin between 80 and 470 K were derived from the fusion enthalpies and heat capacities. The properties determined in this work can be used as reference for developing QSAR relationships, and as input data for calculating solubility or crystallization kinetics.

## Figures and Tables

**Table 1:** Compilation of the experimental data on the fusion enthalpies at melting temperature, $\Delta_{\mathrm{cr}}^{l}H(T_{m})$, of 3EtOAn and Phenac.

Compound	$T_{m}/K$ ^a^	$\Delta_{\mathrm{cr}}^{l}H(T_{m})/{\mathrm{kJ}\;\mathrm{mol}}^{-1}$ ^a^	Ref.
3EtOAn	368.0 ± 0.4	28.9 ± 0.4	[3]
	369.8 ± 0.2	28.1 ± 0.5	This work
	**368.9 ± 1.3**	**28.5 ± 0.6**	**Average ^b^**
Phenac	407.0 ± 0.1	32.0 ± 0.1	[3]
	409.6	30.0 ± 1.0	[7]
	407.0	28.8	[18]
	409.0	31.5	[19]
	407.6	(36.9)	[20]
	408.3	28.8	[21]
	408.1 ± 0.2	32.5 ± 0.2	[22]
	407.7	32.3	[25]
	407.4 ± 0.1	34.1 ± 0.9	[29,30]
	(410.2)	(21.4 ± 0.9)	[31]
	407.2	31.3	[32]
	407.7	30.7	[33]
	408.5 ± 0.2	31.2 ± 0.5	This work
	408.1 ± 0.8	31.2 ± 1.6	Average ^b^

^a^ Uncertainties of the average fusion enthalpy and melting temperature values correspond to the standard deviations of the experimental data points. ^b^ Mean of the literature data and the values obtained in this work.

**Table 2:** Thermodynamic properties of 3EtOAn between 80 and 430 K (*p*° = 0.1 MPa) ^a^.

$\frac{T}{K}$	$\frac{C_{p,m}^{o}}{JK^{-1}mol^{-1}}$	$\frac{\Delta_{80}^{T}S_{m}^{\circ }}{JK^{-1}mol^{-1}}$	$\frac{\Delta_{80}^{T}H_{m}^{\circ }/T}{JK^{-1}mol^{-1}}$	$\frac{-\Delta_{80}^{T}G_{m}^{\circ }/T}{JK^{-1}mol^{-1}}$
Crystal				
80	87.30 ± 0.35	0	0	0
90	95.95 ± 0.38	10.79 ± 0.04	10.19 ± 0.04	0.6016 ± 0.0593
100	104.1 ± 0.4	21.32 ± 0.09	19.17 ± 0.08	2.149 ± 0.115
110	111.6 ± 0.4	31.60 ± 0.13	27.24 ± 0.11	4.361 ± 0.167
120	118.9 ± 0.5	41.63 ± 0.17	34.58 ± 0.14	7.051 ± 0.216
130	125.9 ± 0.5	51.42 ± 0.21	41.33 ± 0.17	10.09 ± 0.26
140	132.7 ± 0.5	61.00 ± 0.24	47.62 ± 0.19	13.38 ± 0.31
150	139.5 ± 0.6	70.39 ± 0.28	53.52 ± 0.21	16.87 ± 0.35
160	146.1 ± 0.6	79.60 ± 0.32	59.10 ± 0.24	20.51 ± 0.40
170	152.7 ± 0.6	88.66 ± 0.35	64.41 ± 0.26	24.25 ± 0.44
180	159.3 ± 0.6	97.58 ± 0.39	69.50 ± 0.28	28.08 ± 0.48
190	166.0 ± 0.7	106.4 ± 0.4	74.40 ± 0.30	31.97 ± 0.52
200	172.8 ± 0.7	115.1 ± 0.5	79.15 ± 0.32	35.90 ± 0.56
210	179.7 ± 0.7	123.7 ± 0.5	83.78 ± 0.34	39.88 ± 0.60
220	186.8 ± 0.7	132.2 ± 0.5	88.30 ± 0.35	43.88 ± 0.64
230	193.9 ± 0.8	140.6 ± 0.6	92.73 ± 0.37	47.90 ± 0.67
240	201.1 ± 0.8	149.0 ± 0.6	97.10 ± 0.39	51.94 ± 0.71
250	208.4 ± 0.8	157.4 ± 0.6	101.4 ± 0.4	55.99 ± 0.75
260	215.8 ± 0.9	165.7 ± 0.7	105.7 ± 0.4	60.05 ± 0.79
270	223.4 ± 0.9	174.0 ± 0.7	109.9 ± 0.4	64.12 ± 0.82
280	231.0 ± 0.9	182.3 ± 0.7	114.1 ± 0.5	68.19 ± 0.86
290	238.8 ± 1.0	190.5 ± 0.8	118.2 ± 0.5	72.27 ± 0.90
298.15	245.3 ± 1.0	197.2 ± 0.8	121.6 ± 0.5	75.59 ± 0.93
300	246.8 ± 1.0	198.7 ± 0.8	122.4 ± 0.5	76.35 ± 0.93
310	254.8 ± 1.0	207.0 ± 0.8	126.5 ± 0.5	80.43 ± 0.97
320	263.0 ± 1.1	215.2 ± 0.9	130.7 ± 0.5	84.51 ± 1.01
330	271.3 ± 1.1	223.4 ± 0.9	134.8 ± 0.5	88.59 ± 1.04
340	279.8 ± 1.1	231.6 ± 0.9	138.9 ± 0.6	92.68 ± 1.08
350	288.4 ± 1.2	239.9 ± 1.0	143.1 ± 0.6	96.77 ± 1.12
360 ^b^	297.0 ± 1.2	248.1 ± 1.0	147.2 ± 0.6	100.9 ± 1.2
368.9 ^b^	304.7 ± 1.2	255.4 ± 1.0	151.0 ± 0.6	104.5 ± 1.2
**Liquid**				
368.9 ^b^	376 ± 11	332.7 ± 2.9	228.2 ± 2.5	104.5 ± 3.8
370 ^b^	377 ± 11	333.8 ± 3.0	228.6 ± 2.5	105.2 ± 3.9
380 ^b^	382 ± 11	343.9 ± 3.3	232.6 ± 2.6	111.3 ± 4.2
390	387 ± 12	353.9 ± 3.6	236.5 ± 2.8	117.4 ± 4.5
400	391 ± 12	363.8 ± 3.9	240.3 ± 2.9	123.5 ± 4.8
410	396 ± 12	373.5 ± 4.1	244.1 ± 3.0	129.4 ± 5.1
420	401 ± 12	383.1 ± 4.4	247.7 ± 3.1	135.4 ± 5.4
430	406 ± 12	392.6 ± 4.7	251.3 ± 3.2	141.2 ± 5.7

^a^ Expanded uncertainties were calculated with a 0.95 level of confidence (*k* ≈ 2). ^b^ The values extrapolated to *T*_m_.

**Table 3:** Thermodynamic properties of Phenac between 80 and 470 K (*p*° = 0.1 MPa) ^a^.

$\frac{T}{K}$	$\frac{C_{p,m}^{o}}{JK^{-1}mol^{-1}}$	$\frac{\Delta_{80}^{T}S_{m}^{\circ }}{JK^{-1}mol^{-1}}$	$\frac{\Delta_{80}^{T}H_{m}^{\circ }/T}{JK^{-1}mol^{-1}}$	$\frac{-\Delta_{80}^{T}G_{m}^{\circ }/T}{JK^{-1}mol^{-1}}$
Crystal				
80	87.63 ± 0.35	0	0	0
90	96.88 ± 0.39	10.86 ± 0.04	10.26 ± 0.04	0.6052 ± 0.0598
100	105.6 ± 0.4	21.52 ± 0.09	19.36 ± 0.08	2.166 ± 0.116
110	113.7 ± 0.5	31.98 ± 0.13	27.57 ± 0.11	4.402 ± 0.169
120	121.4 ± 0.5	42.20 ± 0.17	35.07 ± 0.14	7.128 ± 0.220
130	128.9 ± 0.5	52.22 ± 0.21	42.01 ± 0.17	10.21 ± 0.27
140	136.2 ± 0.5	62.04 ± 0.25	48.48 ± 0.19	13.56 ± 0.31
150	143.3 ± 0.6	71.69 ± 0.29	54.57 ± 0.22	17.12 ± 0.36
160	150.3 ± 0.6	81.16 ± 0.32	60.33 ± 0.24	20.83 ± 0.40
170	157.2 ± 0.6	90.48 ± 0.36	65.83 ± 0.26	24.65 ± 0.45
180	164.0 ± 0.7	99.66 ± 0.40	71.09 ± 0.28	28.56 ± 0.49
190	170.8 ± 0.7	108.7 ± 0.4	76.16 ± 0.30	32.54 ± 0.53
200	177.5 ± 0.7	117.6 ± 0.5	81.06 ± 0.32	36.58 ± 0.57
210	184.4 ± 0.7	126.5 ± 0.5	85.82 ± 0.34	40.65 ± 0.61
220	191.3 ± 0.8	135.2 ± 0.5	90.45 ± 0.36	44.75 ± 0.65
230	198.2 ± 0.8	143.9 ± 0.6	94.99 ± 0.38	48.87 ± 0.69
240	205.2 ± 0.8	152.4 ± 0.6	99.43 ± 0.40	53.00 ± 0.73
250	212.3 ± 0.8	161.0 ± 0.6	103.8 ± 0.4	57.15 ± 0.77
260	219.4 ± 0.9	169.4 ± 0.7	108.1 ± 0.4	61.31 ± 0.80
270	226.7 ± 0.9	177.8 ± 0.7	112.4 ± 0.4	65.47 ± 0.84
280	234.0 ± 0.9	186.2 ± 0.7	116.6 ± 0.5	69.63 ± 0.88
290	241.5 ± 1.0	194.6 ± 0.8	120.8 ± 0.5	73.79 ± 0.92
298.15	247.8 ± 1.0	201.3 ± 0.8	124.1 ± 0.5	77.19 ± 0.95
300	249.3 ± 1.0	202.9 ± 0.8	124.9 ± 0.5	77.96 ± 0.95
310	257.1 ± 1.0	211.2 ± 0.8	129.0 ± 0.5	82.12 ± 0.99
320	265.0 ± 1.1	219.5 ± 0.9	133.2 ± 0.5	86.28 ± 1.03
330	273.2 ± 1.1	227.7 ± 0.9	137.3 ± 0.5	90.45 ± 1.06
340	281.6 ± 1.1	236.0 ± 0.9	141.4 ± 0.6	94.60 ± 1.10
350	290.3 ± 1.2	244.3 ± 1.0	145.5 ± 0.6	98.76 ± 1.14
360	299.4 ± 1.2	252.6 ± 1.0	149.7 ± 0.6	102.9 ± 1.2
370 ^b^	308.6 ± 1.2	260.9 ± 1.0	153.9 ± 0.6	107.1 ± 1.2
380 ^b^	317.8 ± 1.3	269.3 ± 1.1	158.1 ± 0.6	111.2 ± 1.2
390 ^b^	327.0 ± 1.3	277.7 ± 1.1	162.3 ± 0.6	115.4 ± 1.3
400 ^b^	336.1 ± 1.3	286.1 ± 1.1	166.5 ± 0.7	119.6 ± 1.3
408.1 ^b^	343.6 ± 1.4	292.9 ± 1.2	169.9 ± 0.7	122.9 ± 1.4
**Liquid**				
408.1 ^b^	398 ± 12	369.3 ± 5.2	246.4 ± 4.8	122.9 ± 7.1
410 ^b^	399 ± 12	371.2 ± 5.3	247.1 ± 4.8	124.1 ± 7.1
420 ^b^	404 ± 12	380.9 ± 5.6	250.8 ± 4.9	130.1 ± 7.4
430	409 ± 12	390.4 ± 5.9	254.4 ± 5.0	136.0 ± 7.7
440	413 ± 12	399.9 ± 6.2	258.0 ± 5.1	141.9 ± 8.0
450	418 ± 13	409.2 ± 6.4	261.5 ± 5.2	147.7 ± 8.3
460	423 ± 13	418.5 ± 6.7	264.9 ± 5.3	153.5 ± 8.6
470	427 ± 13	427.6 ± 7.0	268.3 ± 5.4	159.3 ± 8.8

^a^ Expanded uncertainties were calculated with a 0.95 level of confidence (*k* ≈ 2). ^b^ The values extrapolated to *T*_m_.

**Table 4:** The experimental and estimated heat capacities of crystal and liquid 3EtOAn and Phenac at 298.15 K.

**Compound A**	$c_{p,m}^{A}$ **(** cr,298.15K **)/J K^−1^ mol^−1^**				
Method	This work	Chickos	Hurst	Goodman (PL)	Goodman (PF)
3EtOAn	245.3 ± 1.0	281.3	252.8	231.6	222.7
Phenac	247.8 ± 1.0	281.3	252.8	231.6	222.7
**Compound A**	$c_{p,m}^{A}$ **(** liq,298.15K **)/J K^−1^ mol^−1^**				
Method	This work		Chickos	Kolska	
3EtOAn	342.6 ± 13.7		329.2	352.6	
Phenac	347.0 ± 13.9		329.2	352.2	

**Table 5:** The experimental and estimated differences between the crystal and liquid heat capacities of 3EtOAn and Phenac and ideal solubilities calculated according to Equation (5) using these differences.

Approach	3EtOAn		Phenac	
	$\frac{\Delta_{cr}^{l}C_{p,m}^{A}}{JK^{-1}mol^{-1}}$	$x_{id}^{A}(298.15K)$	$\frac{\Delta_{cr}^{l}C_{p,m}^{A}}{JK^{-1}mol^{-1}}$	$x_{id}^{A}(298.15K)$
$\Delta_{\mathrm{cr}}^{l}c_{p,m}^{A}=0$	0	0.110	0	0.034
Pappa, Ia ^a^	109.0	0.151	106.9	0.068
Pappa, Ib ^a^	116.9	0.155	114.6	0.072
Pappa, II ^a^	77.3	0.138	76.5	0.056
Wu ^b^	49.5	0.127	49.5	0.047
This work (*T*_m_) ^c^	71.8	0.136	54.9	0.048
This work ^d^	212.2–0.381 (*T*/K)	0.140	238.2–0.449 (*T*/K)	0.054

^a^ Approaches for $\Delta_{\mathrm{cr}}^{l}c_{p,m}^{A}$ estimation described in Ref. [44]. ^b^ Approach for $\Delta_{\mathrm{cr}}^{l}c_{p,m}^{A}$ estimation described in Ref. [55]. ^c^ $\Delta_{\mathrm{cr}}^{l}c_{p,m}^{A}$ (T_m_) measured in this work. The combined uncertainty is ±14.0 J K^−1^ mol^−1^. ^d^ $\Delta_{\mathrm{cr}}^{l}c_{p,m}^{A}$ (T) calculated using Equations (1)–(4). The combined uncertainty is ±14.0 J K^−1^ mol^−1^.

**Table 6:** Fusion enthalpies of 3EtOAn and Phenac at *T*_m_ (column 2) and at 298.15, calculated using Kirchhoff’s law (column 3) and obtained by solution calorimetry (column 4).

Compound A	$\Delta_{\mathrm{cr}}^{l}H^{A}(T_{m})$/kJ mol^−1^	$\Delta_{\mathrm{cr}}^{l}H^{A}(298.15)$/kJ mol^−1^	
Method	DSC	Kirchhoff’s law ^a^	solution calorimetry
3EtOAn	28.5 ± 0.6	22.4 ± 1.2	23.3 ± 1.0
Phenac	31.2 ± 1.6	22.5 ± 2.2	24.9 ± 1.0

^a^ Calculated from $\Delta_{\mathrm{cr}}^{l}H(T_{m})$ (second column) and the experimental values of *C*_p,m_(cr, *T*) and *C*_p,m_(l, *T*), using Equation (6). Uncertainties correspond to propagated errors of the sums of $\Delta_{\mathrm{cr}}^{l}H(T_{m})$ and $\int_{T_{m}}^{298.15}\Delta_{\mathrm{cr}}^{l}c_{p,m}^{A}(T)\;dT$ uncertainties.

## Data Availability

All data are available on request from the corresponding author.

## Supplementary Materials

The following supporting information can be downloaded at: https://www.mdpi.com/article/10.3390/molecules28207027/s1, Figure S1: The heat flow rates during the measurements of the heat capacity of the crystalline (a) and liquid (b) 3EtOAn and the crystalline (c) and liquid (d) Phenac by DSC; Figure S2: The heat flow rates during the measurements of the fusion enthalpy of 3EtOAn (a) and Phenac (b) measured by DSC; Figure S3: The heat flow rates of the crystalline (a) and liquid (b) 3EtOAn sample and the crystalline (c) and liquid (d) Phenac sample measured using FSC; Figure S4: Relative deviations of the experimental heat capacities of the 3-ethoxyacetanilide from the approximated values in the temperature range of (80–350) K for crystal (adiabatic calorimetry); Figure S5: Relative deviations of the experimental heat capacities of 3-ethoxyacetanilide from the approximated values in the temperature range of (290–430) K; Figure S6: Relative deviations of the experimental heat capacities of phenacetin from the approximated values in the temperature range of (80–370) K; Figure S7: Relative deviations of the experimental heat capacities of phenacetin from the approximated values in the temperature range of (290–480) K; Table S1: Provenance and purity of the materials; Table S2: Enthalpies of fusion of 3-ethoxyacetanilide and phenacetin at 0.1 MPa measured by DSC in this work; Table S3: Experimental heat capacities of 3-ethoxyacetanilide at saturation pressure measured by adiabatic calorimetry; Table S4: Experimental heat capacities of phenacetin at saturation pressure measured by adiabatic calorimetry; Table S5: Experimental heat capacities of crystal, supercooled liquid, and liquid 3-ethoxyacetanilide and phenacetin determined in this work at 0.1 MPa by DSC and FSC; Table S6: Experimental solution enthalpies of 3-ethoxyacetanilide and phenacetin in DMF measured in this work at 298.15 K and 0.1 MPa; Table S7: Coefficients of the approximating equation used for calculation of the thermodynamic properties of 3-ethoxyacetanilide in the different temperature ranges, the number of points used for fitting, and root-mean-square error RMSE; Table S8: Coefficients of the approximating equation used for calculation of the thermodynamic properties of phenacetin in the different temperature ranges, the number of points used for fitting, and root-mean-square error RMSE.

## Sample Availability

Samples are not available from the authors.
