# Supplementary material for: Thermodynamic Properties of 3- and 4-Ethoxyacetanilides between 80 and 480 K
Source: Molecules. 2023 Oct 11;28(20):7027. doi: 10.3390/molecules28207027 (PMC10609036; doi:10.3390/molecules28207027)
Supplement: Supplementary file 1 [file molecules-28-07027-s001.zip › molecules-2620180-supplementary.pdf]

Table S1. Provenance and purity of the materials.

Chemical Name	CASRN	Source	Initial mass fraction purity [a]
3-ethoxyacetanilide	591-33-3	Alfa	0.998
phenacetin	62-44-2	Alfa	0.998
N,N-dimethylformamide	68-12-2	Aldrich	0.999 [b]
anthracene	120-12-7	Aldrich	0.999
benzoic acid	65-85-0	Aldrich	0.999
biphenyl	92-52-4	Aldrich	0.999
indium	7440-74-6	PE	0.99999
zinc	7440-66-6	PE	0.99999
sapphire	1344-28-1	PE	not provided
bismuth	7440-69-9	Netzsch	0.99999

[a] Mass fraction purity stated by supplier.

[b] Mass fraction of water determined by Karl Fisher titration was 0.0003.

Table S2. Enthalpies of fusion of 3-ethoxyacetanilide and phenacetin at 0.1 MPa measured by DSC in this work.

Compound	$\frac{m}{\text{mg}}$	$\frac{T_m}{\text{K}}$	$\frac{\Delta_{cr}^{l}H(T_m)}{\text{kJ mol}^{-1}}$
3-ethoxyacetanilide	7.98	369.8	27.94
		369.6	28.17
	11.46	369.7	27.94
		370.2	28.27
	**Average [a]**	**369.8±0.3**	**28.08±0.45**
phenacetin	9.51	408.5	31.24
		408.3	31.49
	6.36	408.8	30.99
		408.5	31.23
	**Average [a]**	**408.5±0.2**	**31.24±0.52**

[a] The uncertainties reported inside Table 2 correspond to the expanded uncertainty of the mean *U* (0.95 level of confidence, coverage factor of 2.0) and include the reproducibility of the measurements and calibration (0.1 K for temperature and 1 % for enthalpy, see Sec. 4.3); the standard uncertainty of pressure $u(p)$ = 5 kPa.

Table S3. Experimental heat capacities of 3-ethoxyacetanilide at saturation pressure measured by adiabatic calorimetry.

$\frac{\langle T \rangle}{K}$	$\frac{C_{s,m}{}^{a}}{J \cdot K^{-1} \cdot mol^{-1}}$	$\frac{\langle T \rangle}{K}$	$\frac{C_{s,m}{}^{a}}{J \cdot K^{-1} \cdot mol^{-1}}$	$\frac{\langle T \rangle}{K}$	$\frac{C_{s,m}{}^{a}}{J \cdot K^{-1} \cdot mol^{-1}}$
79.16	86.56	139.55	132.4	201.58	173.9
81.08	88.27	141.54	133.7	203.60	175.3
82.99	89.91	143.52	135.1	205.61	176.7
84.91	91.64	145.51	136.4	207.64	178.0
86.83	93.27	147.49	137.7	209.66	179.5
88.75	94.90	149.48	139.1	211.68	180.9
90.68	96.49	151.47	140.4	213.70	182.4
92.60	98.11	153.46	141.8	215.73	183.8
94.53	99.72	155.45	143.1	217.75	185.2
96.47	101.3	157.44	144.4	219.77	186.6
98.40	102.8	159.44	145.8	221.80	188.0
100.34	104.3	161.43	147.1	223.83	189.5
102.28	105.9	163.43	148.4	225.85	191.0
104.21	107.4	165.43	149.7	227.88	192.3
106.16	108.8	167.43	151.0	229.91	193.8
108.10	110.2	169.43	152.4	231.94	195.3
110.06	111.7	171.43	153.7	233.97	196.6
112.01	113.1	173.43	155.0	236.00	198.2
113.96	114.6	175.44	156.3	238.02	199.7
115.92	116.0	177.44	157.6	240.05	201.2
117.88	117.4	179.45	159.0	242.08	202.6
119.84	118.8	181.46	160.3	244.11	204.0
121.80	120.2	183.47	161.7	246.14	205.5
123.76	121.5	185.48	162.9	248.18	207.0
125.73	122.9	187.48	164.3	250.21	208.6
127.70	124.3	189.49	165.7	252.24	210.1
129.67	125.7	191.50	167.0	254.27	211.6
131.64	127.0	193.51	168.4	256.30	213.1
133.62	128.4	195.52	169.7	258.33	214.6
135.59	129.7	197.54	171.1	260.37	216.1
137.57	131.1	199.56	172.5	262.40	217.7

$\langle T\rangle$/K	$C_{s,m}$ [a]/J·K^{-1}·mol^{-1}	$\langle T\rangle$/K	$C_{s,m}$ [a]/J·K^{-1}·mol^{-1}	$\langle T\rangle$/K	$C_{s,m}$ [a]/J·K^{-1}·mol^{-1}
264.43	219.2	301.01	247.6	337.70	277.7
266.46	220.6	303.05	249.2	339.74	279.5
268.50	222.3	305.08	250.9	341.78	281.4
270.52	223.7	307.12	252.5	343.83	283.1
272.56	225.4	309.15	254.3	345.88	284.8
274.59	226.8	311.19	255.9	347.92	286.6
276.62	228.4	313.23	257.4	349.97	288.5
278.66	230.0	315.26	259.1	352.02	290.4
280.69	231.6	317.30	260.7	354.06 [b]	292.7
282.72	233.1	319.34	262.3	356.11 [b]	294.9
284.76	234.7	321.37	264.2	358.16 [b]	297.6
286.78	236.3	323.41	265.9	360.20 [b]	301.6
288.82	237.9	325.45	267.6	362.24 [b]	308.9
290.85	239.4	327.49	269.2	364.27 [b]	320.1
292.88	241.1	329.53	270.8	366.24 [b]	364.8
294.92	242.7	331.57	272.5	367.92 [b]	707.2
296.95	244.3	333.61	274.4	368.87 [b]	4104
298.98	245.9	335.65	276.0		

[a] Average heat capacity at the mean temperature of an experiment. The combined expanded uncertainties are $U_c(T) = 0.01$ K, $U_c(C_{s,m}) = 0.004C_{s,m}$ for 0.95 level of confidence ($k \approx 2$).

[b] Limitations on the upper temperature limit of measurements of the adiabatic calorimeter (370 K) make it possible to fix only part of the ascending branch of the melting curve and do not allow determining the enthalpy of melting of the substance; therefore, these values weren't used in the further calculations.

Table S4. Experimental heat capacities of phenacetin at saturation pressure measured by adiabatic calorimetry.

$\langle T\rangle$/K	$C_{s,m}$ [a]/J·K^{-1}·mol^{-1}	$\langle T\rangle$/K	$C_{s,m}$ [a]/J·K^{-1}·mol^{-1}	$\langle T\rangle$/K	$C_{s,m}$ [a]/J·K^{-1}·mol^{-1}
78.88	86.56	84.66	91.99	90.43	97.24
80.84	88.43	86.58	93.80	92.36	99.00
82.75	90.26	88.50	95.51	94.29	100.7

$\frac{\langle T \rangle}{\mathrm{K}}$	$\frac{C_{s,\mathrm{m}}{}^{\mathrm{a}}}{\mathrm{J \cdot K^{-1} \cdot mol^{-1}}}$	$\frac{\langle T \rangle}{\mathrm{K}}$	$\frac{C_{s,\mathrm{m}}{}^{\mathrm{a}}}{\mathrm{J \cdot K^{-1} \cdot mol^{-1}}}$	$\frac{\langle T \rangle}{\mathrm{K}}$	$\frac{C_{s,\mathrm{m}}{}^{\mathrm{a}}}{\mathrm{J \cdot K^{-1} \cdot mol^{-1}}}$
96.22	102.4	161.32	151.2	227.93	196.8
98.16	104.0	163.32	152.6	229.97	198.2
100.10	105.6	165.32	154.0	232.00	199.5
102.04	107.3	167.32	155.3	234.04	201.0
103.99	108.9	169.32	156.7	236.08	202.4
105.93	110.5	171.33	158.1	238.11	204.0
107.88	112.0	173.34	159.5	240.15	205.4
109.84	113.6	175.35	160.8	242.19	206.7
111.80	115.1	177.36	162.2	244.23	208.2
113.75	116.6	179.37	163.6	246.26	209.7
115.71	118.2	181.38	165.0	248.30	211.2
117.67	119.7	183.39	166.3	250.34	212.5
119.63	121.2	185.40	167.6	252.38	214.0
121.60	122.7	187.42	169.0	254.42	215.4
123.56	124.2	189.44	170.4	256.46	216.9
125.54	125.7	191.45	171.7	258.50	218.4
127.51	127.1	193.46	173.1	260.54	219.7
129.48	128.6	195.48	174.5	262.58	221.3
131.46	130.0	197.50	175.8	264.62	222.8
133.44	131.5	199.51	177.2	266.66	224.3
135.42	132.9	201.53	178.6	268.70	225.7
137.41	134.3	203.56	179.9	270.74	227.3
139.39	135.8	205.58	181.4	272.78	228.6
141.38	137.2	207.61	182.7	274.82	230.1
143.37	138.6	209.64	184.1	276.86	231.7
145.35	140.0	211.67	185.5	278.91	233.2
147.34	141.5	213.70	186.9	280.95	234.7
149.33	142.9	215.73	188.3	282.99	236.2
151.33	144.3	217.76	189.7	285.03	237.7
153.33	145.7	219.79	191.1	287.07	239.3
155.33	147.1	221.83	192.6	289.11	240.7
157.32	148.5	223.86	194.0	291.16	242.4
159.32	149.9	225.90	195.4	293.20	244.0

$\frac{\langle T \rangle}{\text{K}}$	$\frac{C_{s,\text{m}}{}^{\text{a}}}{\text{J·K}^{-1}\text{·mol}^{-1}}$	$\frac{\langle T \rangle}{\text{K}}$	$\frac{C_{s,\text{m}}{}^{\text{a}}}{\text{J·K}^{-1}\text{·mol}^{-1}}$	$\frac{\langle T \rangle}{\text{K}}$	$\frac{C_{s,\text{m}}{}^{\text{a}}}{\text{J·K}^{-1}\text{·mol}^{-1}}$
295.24	245.5	319.77	264.7	344.37	285.2
297.28	247.1	321.82	266.6	346.42	287.1
299.32	248.6	323.87	268.2	348.47	289.0
301.37	250.2	325.91	270.0	350.52	290.6
303.41	251.8	327.96	271.5	352.58	292.6
305.45	253.4	330.01	273.2	354.64	294.4
307.50	255.2	332.06	274.9	356.69	296.2
309.54	256.8	334.11	276.6	358.74	298.2
311.59	258.3	336.16	278.4	360.79	300.2
313.63	260.0	338.21	280.2	362.84	302.2
315.68	261.6	340.26	281.8	364.90	303.9
317.72	263.2	342.31	283.6	366.95	305.9

[a] Average heat capacity at the mean temperature of an experiment. The combined expanded uncertainties are $U_c(T) = 0.01$ K, $U_c(C_{s,\text{m}}) = 0.004C_{s,\text{m}}$ for 0.95 level of confidence ($k \approx 2$)

Table S5. Experimental heat capacities of crystal, supercooled liquid, and liquid 3-ethoxyacetanilide and phenacetin determined in this work at 0.1 MPa by DSC [a] and FSC [b].

3-ethoxyacetanilide		phenacetin	
$\frac{T}{\text{K}}$	$\frac{C_{\text{p,m}}(T)}{\text{J}\cdot\text{K}^{-1}\cdot\text{mol}^{-1}}$	$\frac{T}{\text{K}}$	$\frac{C_{\text{p,m}}(T)}{\text{J}\cdot\text{K}^{-1}\cdot\text{mol}^{-1}}$
Crystal		Crystal	
330	264.6±7.9	330	265.9±8.0
335	269.1±8.1	335	270.6±8.1
340	273.4±8.2	340	275.1±8.3
350	282.6±8.5	345	279.5±8.4
355	287.6±8.6	350	284.2±8.5
Supercooled liquid		355	289.1±8.7
290	336.3±13.5	360	294.3±8.8
295	339.5±13.6	365	299.3±9.0
300	343.1±13.7	370	304.8±9.1
305	346.5±13.9	375	310.3±9.3

310	349.3±14.0	380	316.1±9.5
315	351.7±14.1	Supercooled liquid	
320	353.2±14.1	290	342.8±13.7
325	355.9±14.2	295	345.3±13.8
330	357.9±14.3	300	347.5±13.9
335	360.8±14.4	305	350.0±14.0
Liquid		Liquid	
385	386.1±11.6	430	410.5±12.3
390	387.7±11.6	435	412.5±12.4
395	389.6±11.7	440	414.5±12.4
400	391.8±11.8	445	416.5±12.5
405	393.9±11.8	450	418.4±12.6
410	396.0±11.9	455	420.4±12.6
415	398.0±11.9	460	422.4±12.7
420	399.9±12.0	465	424.4±12.7
425	402.1±12.1	470	426.4±12.8
430	404.6±12.1	475	428.3±12.9

[a] Average experimental heat capacities interpolated every 5 K. The combined expanded uncertainties are $U_c(T) = 0.1$ K, $U_c(C_{p,m}) = 0.03c_p$ for 0.95 level of confidence ($k \approx 2$)

[b] Average experimental heat capacities interpolated every 5 K. The combined expanded uncertainties are $U_c(T) = 0.1$ K, $U_c(C_{p,m}) = 0.04c_p$ for 0.95 level of confidence ($k \approx 2$)

Table S6. Experimental solution enthalpies of 3-ethoxyacetanilide and phenacetin in DMF measured in this work at 298.15 K and 0.1 MPa [a].

Solute	Mass of sample [b] / mg	Molality [c] / mmol kg^{-1}	$\Delta_{soln}H$ / kJ mol^{-1}
3-ethoxyacetanilide	32.5	2.25	19.17
	42.0	5.16	18.73
	41.9	8.06	19.32
	33.4	10.38	18.77
	33.0	12.66	19.10
			$\Delta_{soln}H = 19.02 \pm 0.23$ kJ mol^{-1} [d]
phenacetin	33.9	2.35	20.56
	35.2	4.79	20.61
	36.3	7.30	20.52

37.2	9.87	20.69
37.8	12.49	20.62

$\Delta_{\text{soln}}H = 20.60 \pm 0.06$ kJ mol^{-1} [d]

[a] Standard uncertainties *u* are *u(T)* = 0.01 K, *u(p)* = 5 kPa.

[b] Mass of solute sample which was added in each dissolution experiment.

[c] Molality of solute in solution after experiments. Standard uncertainty *u(b)* = 0.01 mmol kg^{-1}.

[d] Average enthalpy of solution. Uncertainties reported inside Table S6 correspond to the combined expanded uncertainties of the mean *U* (0.95 level of confidence, coverage factor 2.0) and include the reproducibility of the measurement and calibration (0.1 %).

Table S7. Coefficients of the approximating equation $C^{\circ}_{\text{p,m}}$ / J mol^{-1} K^{-1} $= \sum_{n=0}^{3} a_n (T/K)^n$ used for calculation of the thermodynamic properties of 3-ethoxyacetanilide in the different temperature ranges, the number of points used for fitting and root-mean-square error *RMSE* / (J mol^{-1} K^{-1}).

Temperature range	a_0	a_1	$a_2 \cdot 10^4$	$a_3 \cdot 10^6$	N	*RMSE*
			Crystal			
79.16 – 104.5	–4.9452	1.4472	–40.995	5.2912	21	0.017
104.5 – 197.5	5.0034	1.2948	–37.872	7.5402	55	0.028
197.5 – 306.9	43.905	0.63889	–1.6433	0.96236	61	0.046
306.9 – 347.4	88.912	0.26374	8.7558		27	0.089
347.4 – 368.9	–12.116	0.85872			14	0.13
			Liquid			
298 – 430	200.14	0.47799			94	0.96

Table S8. Coefficients of the approximating equation $C^{\circ}_{\text{p,m}}$ / J mol^{-1} K^{-1} $= \sum_{n=0}^{3} a_n (T/K)^n$ used for calculation of the thermodynamic properties of phenacetin in the different temperature ranges, the number of points used for fitting and root-mean-square error *RMSE* / (J mol^{-1} K^{-1}).

Temperature range	a_0	a_1	$a_2 \cdot 10^3$	$a_3 \cdot 10^6$	N	*RMSE*
			Crystal			
78.88 – 107.6	–11.562	1.5708	–4.6964	7.0096	21	0.017
107.6 – 202.3	2.9738	1.2958	–3.2555	5.7023	53	0.023
202.3 – 290.0	25.526	0.92619	–1.2882	2.2850	51	0.051
290.0 – 363.18	–195.79	3.0196	–7.8877	9.2244	40	0.076

363.18 – 408.1	–30.877	0.91756		11	0.12
Liquid					
290 – 470	207.28	0.46853		65	0.92

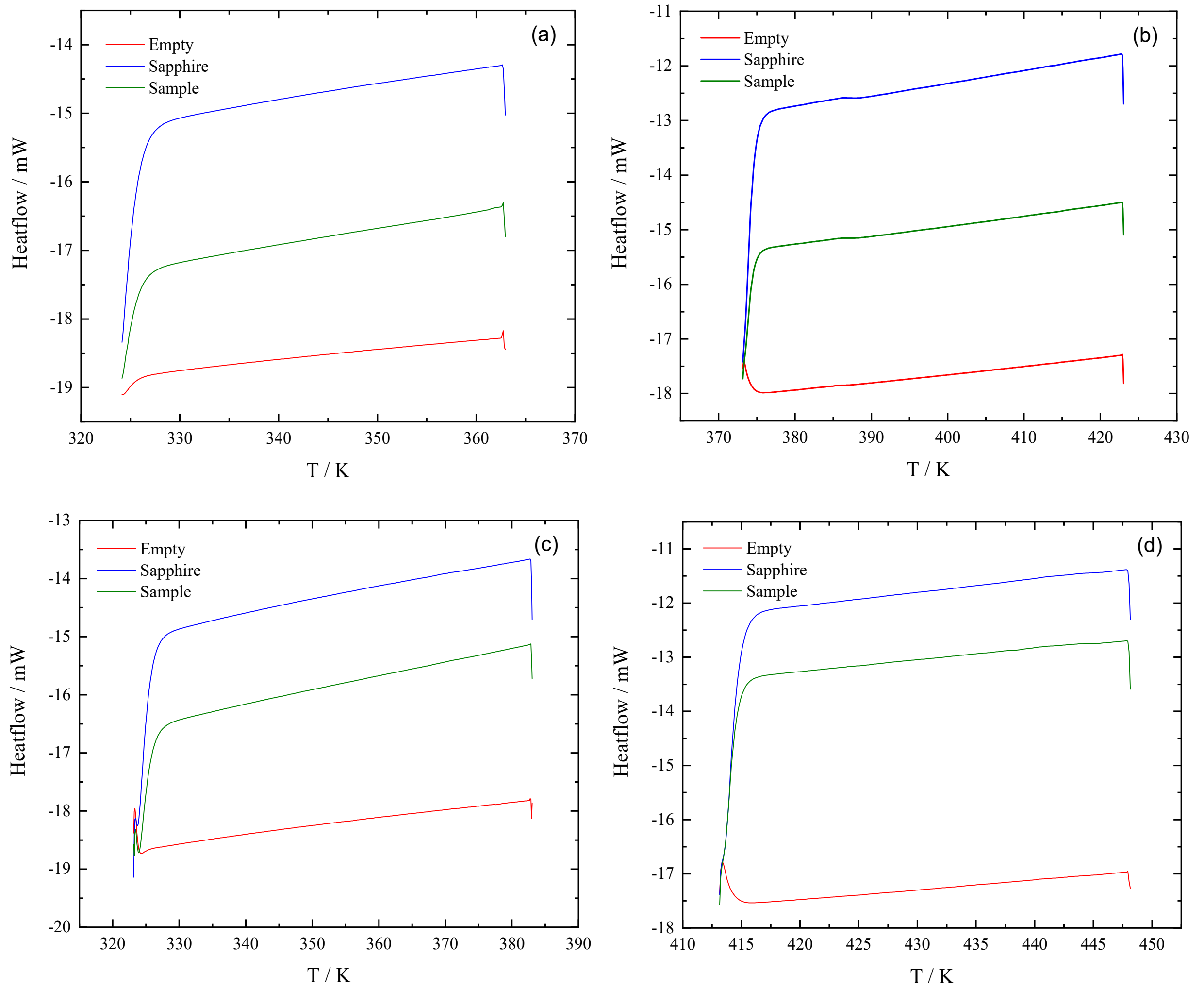

Figure S1. The heat flow rates during the measurements of the heat capacity of the crystalline (a) and liquid (b) 3EtOAn and the crystalline (c) and liquid (d) Phenac by DSC.

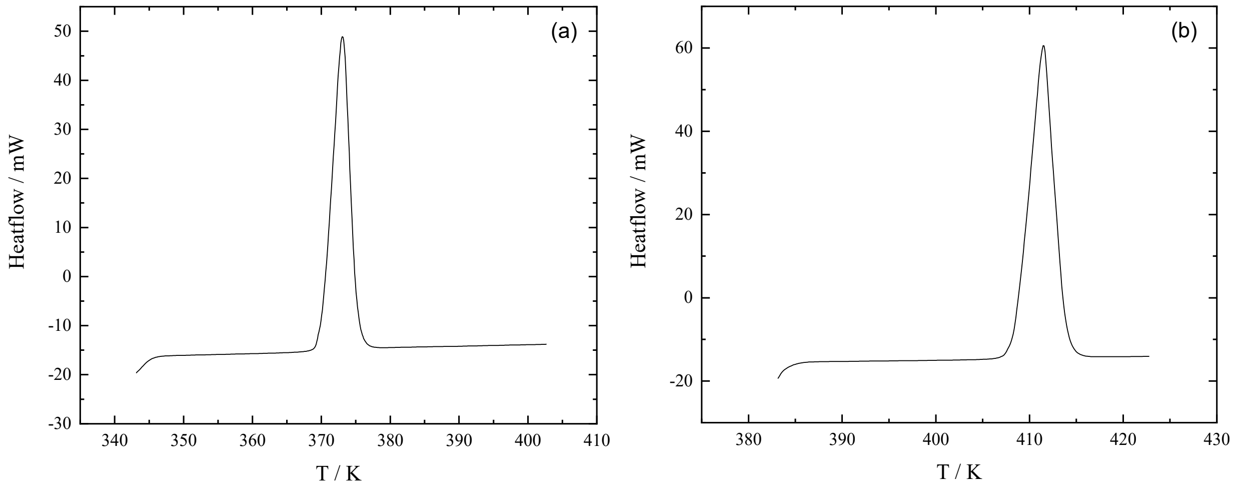

Figure S2. The heat flow rates during the measurements of the fusion enthalpy of 3EtOAn (a) and Phenac (b) measured by DSC.

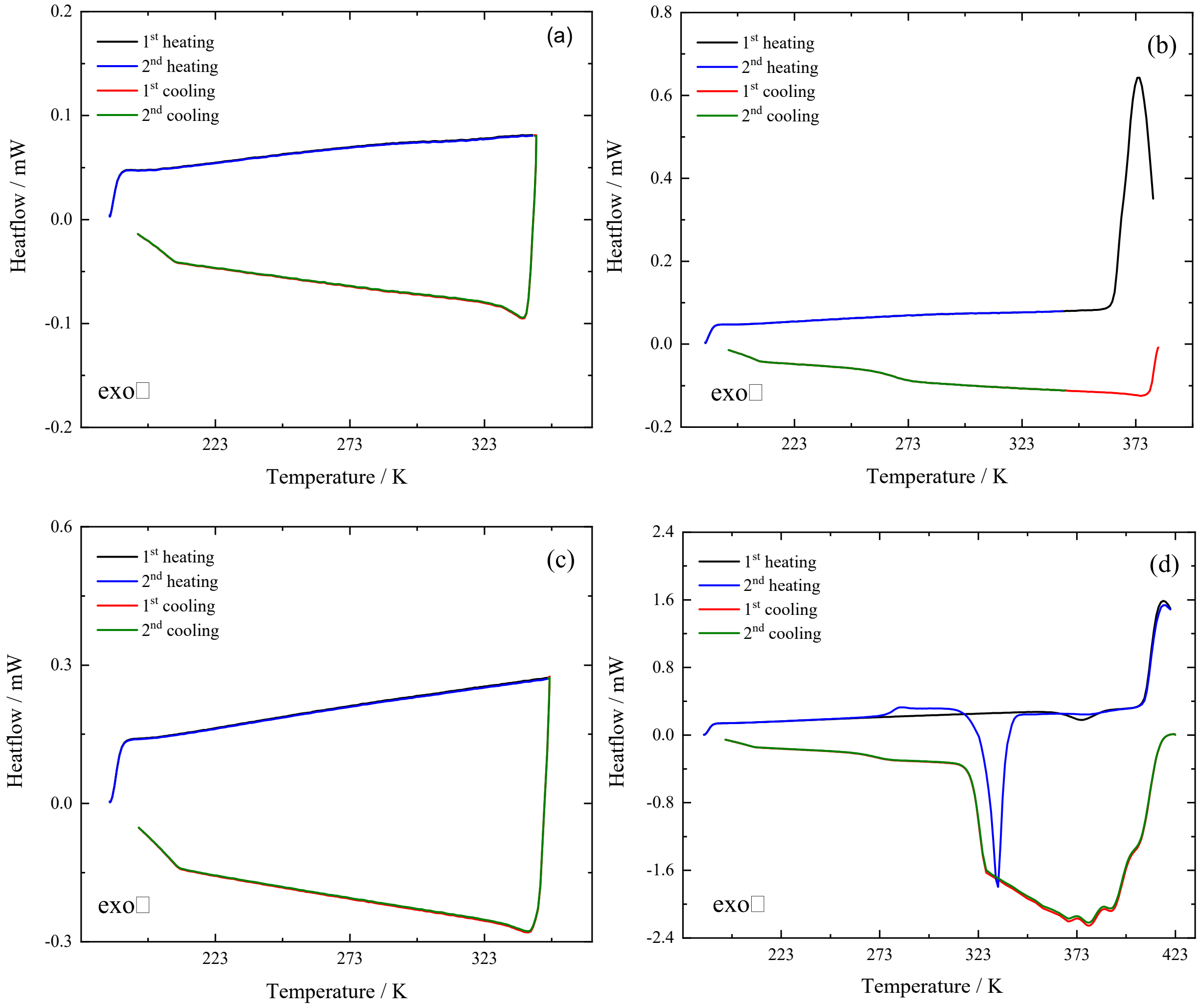

Figure S3. The heat flow rates to the crystalline (a) and liquid (b) 3EtOAn sample and the crystalline (c) and liquid (d) Phenac sample measured using FSC.

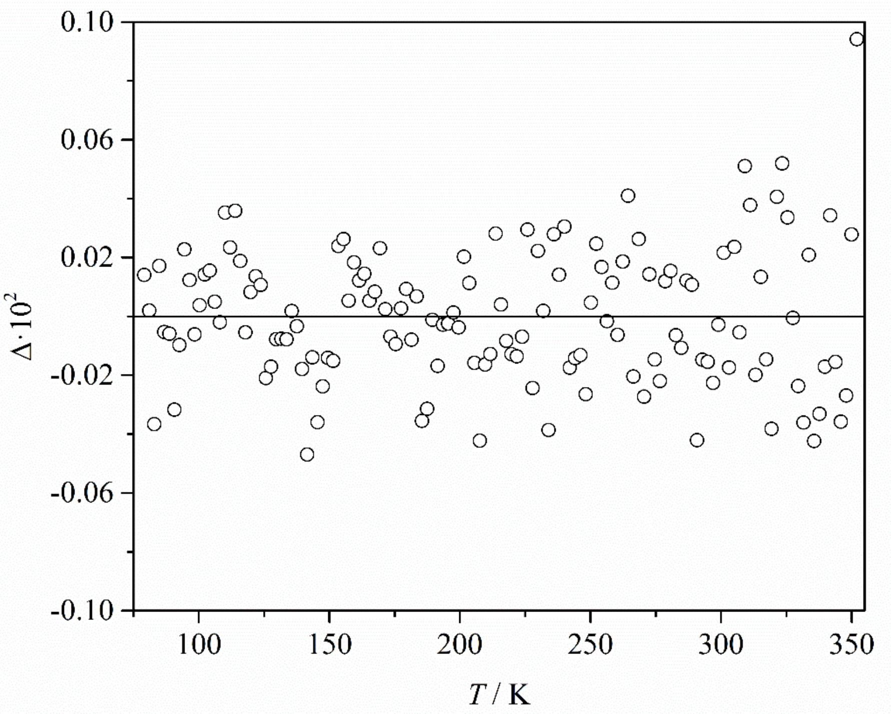

Figure S4. Relative deviations $\Delta = C_{p,m}(\exp)/C_{p,m}(\text{smoothed}) - 1$ of the experimental heat capacities of the 3-ethoxyacetanilide from the approximated values in the temperature range of (80 – 350) K for crystal (adiabatic calorimetry).

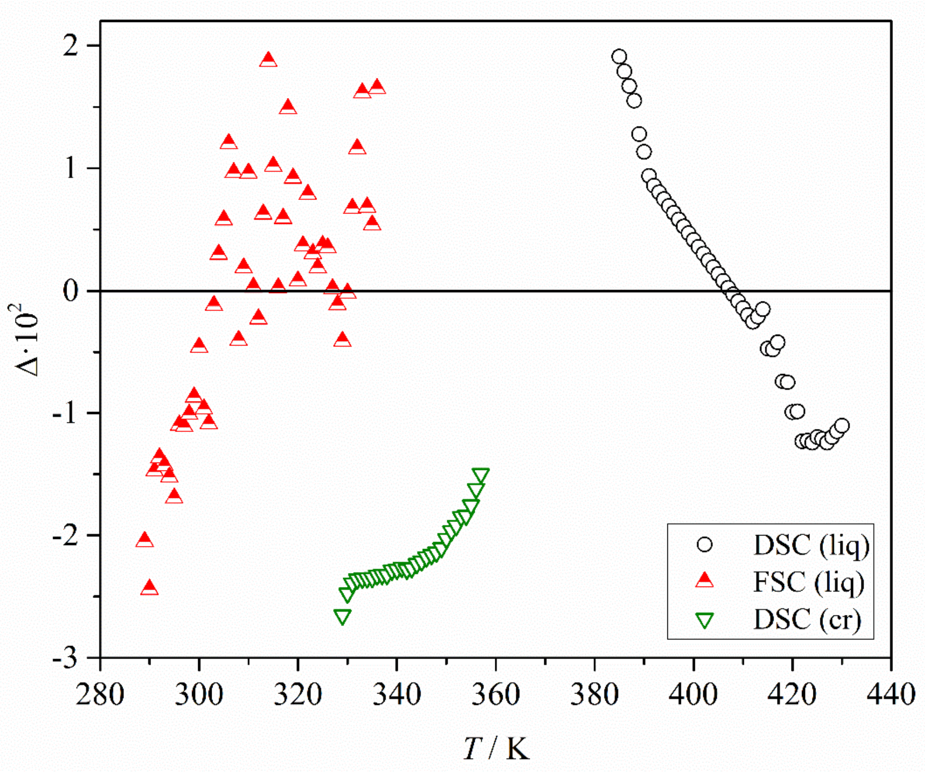

Figure S5. Relative deviations $\Delta = C_{p,m}(\exp)/C_{p,m}(\text{smoothed}) - 1$ of the experimental heat capacities of the 3-ethoxyacetanilide from the approximated values in the temperature range of (290 – 430) K.

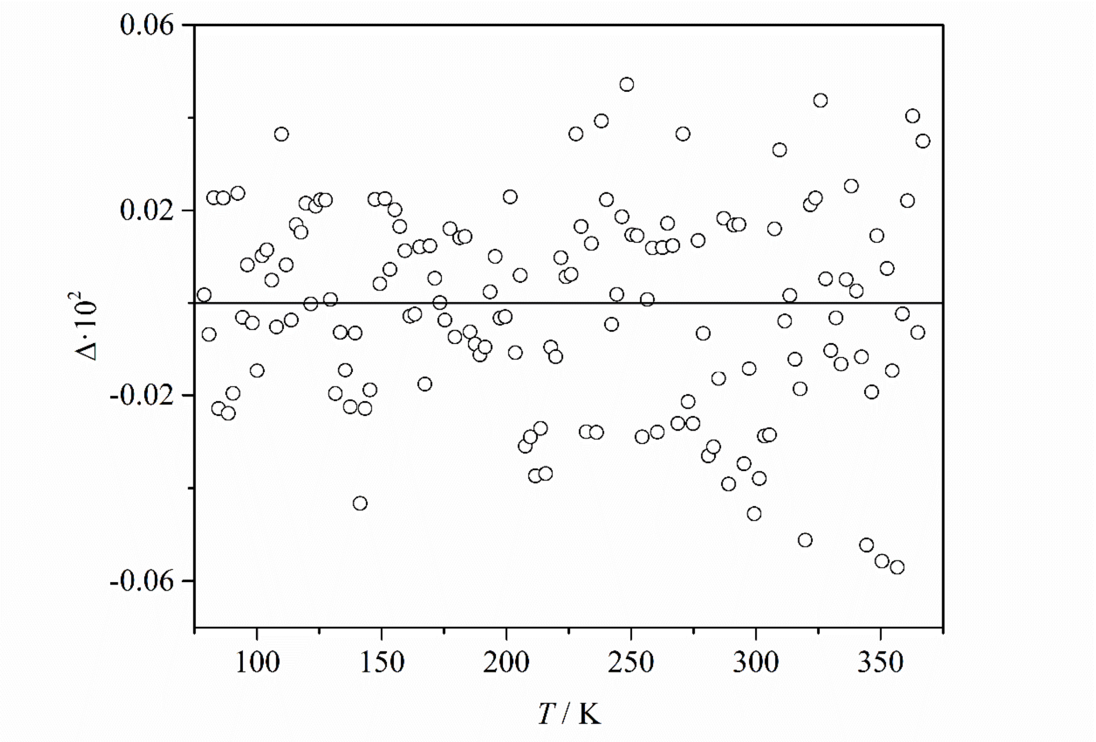

Figure S6. Relative deviations $\Delta = C_{p,m}(\exp)/C_{p,m}(\text{smoothed}) - 1$ of the experimental heat capacities of the phenacetin from the approximated values in the temperature range of (80 – 370) K.

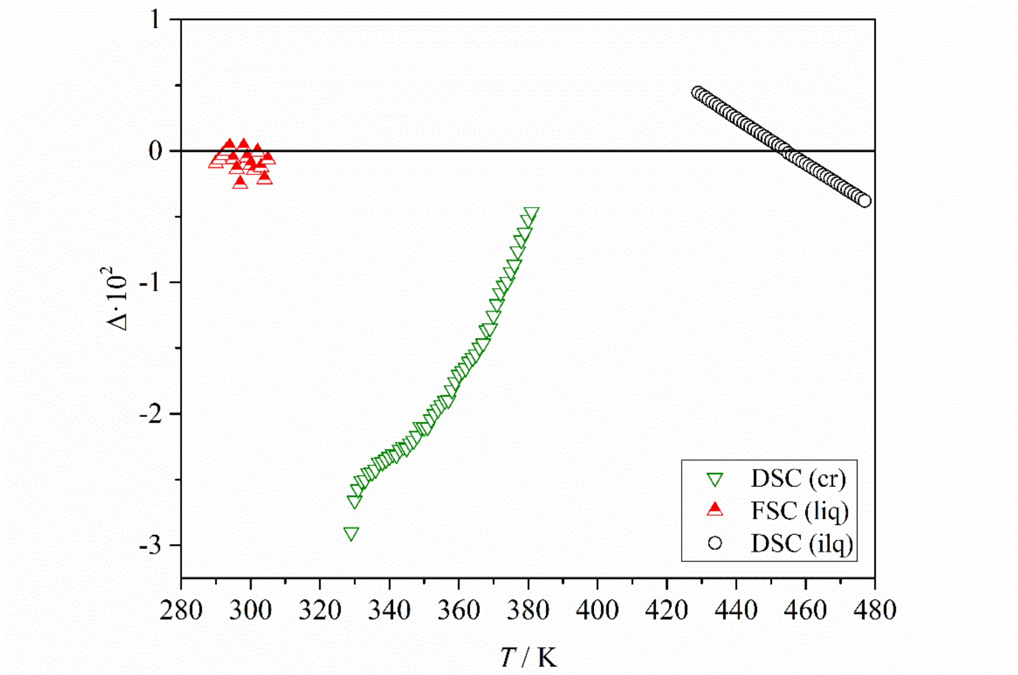

Figure S7. Relative deviations $\Delta = C_{p,m}(\exp)/C_{p,m}(\text{smoothed}) - 1$ of the experimental heat capacities of the phenacetin from the approximated values in the temperature range of (290 – 480) K.
